# MHC Class I Chain-Related Gene A Diversity in Patients with Cutaneous Malignant Melanoma from Southeastern Spain

**DOI:** 10.1155/2015/831864

**Published:** 2015-03-09

**Authors:** José Antonio Campillo, Ruth López-Hernández, Helios Martínez-Banaclocha, José Miguel Bolarín, Lourdes Gimeno, Anna Mrowiec, Manuela López, Beatriz Las Heras, Alfredo Minguela, Maria Rosa Moya-Quiles, Isabel Legáz, José Francisco Frías-Iniesta, Ana María García-Alonso, María Rocío Álvarez-López, Jorge Antonio Martínez-Escribano, Manuel Muro

**Affiliations:** ^1^Immunology Department, University Clinical Hospital Virgen de la Arrixaca, Carretera Madrid-Cartagena, El Palmar, 30120 Murcia, Spain; ^2^Department of Social and Health Sciences, Area of Legal and Forensic Medicine, University of Murcia, 30100 Murcia, Spain; ^3^Dermatology Department, University Clinical Hospital Virgen de la Arrixaca, Carretera Madrid-Cartagena, El Palmar, 30120 Murcia, Spain

## Abstract

A limited number of studies have been performed so far on the polymorphism in the transmembrane region (exon 5) of the major histocompatibility complex class I chain-related gene A (MICA) in patients with melanoma. However, the influence of MICA polymorphism in extracellular domains (exons 2, 3, and 4) has not been investigated on melanoma disease. This study aims to characterize the influence of extracellular MICA polymorphism, and its previously described linkage disequilibrium with HLA-B locus, on patients with cutaneous melanoma from southeastern Spain. For this purpose, MICA and HLA-B genotyping was performed in 233 patients and 200 ethnically matched controls by luminex technology. Patients were classified according to the presence of methionine or valine at codon 129 of MICA gene. We found a high frequency of MICA^*^009 in melanoma patients compared with controls (*P* = 0.002, Pc = 0.03). Our results also showed an association between MICA^*^009 and HLA-B^*^51 alleles in both patients and controls. This association was stronger in patients than controls (*P* = 0.015). However, a multivariate logistic regression model showed that neither MICA^*^009 nor the combination MICA^*^009/HLA-B^*^51 was associated with melanoma susceptibility. No relationship was observed between MICA-129 dimorphism and melanoma nor when MICA polymorphism was evaluated according to clinical findings at diagnosis.

## 1. Introduction

Natural killer (NK) and antigen-specific cytolytic CD8^+^ T cells are believed to be involved in the immune response against melanoma [1–3], a tumor characterized by a poor clinical outcome [4]. Although melanoma is considered one of the most immunogenic tumors [2], it can escape from the host immune surveillance, especially if a state of tolerance is induced [5–7].

The effector function of NK and CD8^+^ T cells may be regulated by a balance between stimulatory and suppressing signals. These signals are mediated by a group of receptors after the interaction with their cognate ligands expressed on tumor cells [8–10], including tumoral melanocytes [1, 11]. In this context, it should be noted that killing of melanoma cells by NK cells is controlled by multiple activating receptor-ligand interactions [3, 12, 13], of which the NKG2D/NKG2D ligand system seems to be the major entity responsible for the lysis of melanoma tumor cells [3].

In humans, ligands of NKG2D activating receptor include the major histocompatibility complex class I chain-related (MIC) and UL16 binding protein molecules [14–16]. MIC genes include seven members (MICA-MICG), five of which are pseudogenes and the other two (MICA and MICB) are functional genes closely related to each other [15]. Unlike human leukocyte antigen (HLA) classical class I genes, MICA/B genes encode for stress-induced cell-surface molecules which do not associate with *β*_2_-microglobulin and do not seem to present peptides [17]. MICA/B proteins have a limited tissue distribution [17, 18], as they are mainly expressed in gastrointestinal epithelium, keratinocytes, endothelial cells, monocytes and fibroblasts, renal and pancreatic allograft, and lesions and cell lines derived from both epithelial tumors and cutaneous melanoma [1, 11, 19].

MICA gene is located in the HLA class I region of chromosome 6, 46 kb centromeric to the HLA-B locus, and is by far the most divergent mammalian MIC gene known [20]. It displays a high degree of allelic polymorphism in exon 5, encoding the transmembrane region [21], and in exons 2, 3, and 4, encoding the three extracellular domains alfa-1, alfa-2, and alfa-3 of the MICA protein, respectively [22]. Although the functional significance of MICA gene diversity is unknown, certain changes in the amino acid sequence of MICA proteins could influence the affinity with its cognate NKG2D receptor [23]. Thus, it has been demonstrated that the presence of methionine (Met) or valine (Val) at codon 129 of the alfa-2 domain may confer strong or weak affinity, respectively.

MICA polymorphism has been associated with a variety of diseases [24], including inflammatory and autoimmune diseases [25, 26], infectious and tumor diseases [27, 28], or even chronic graft-versus-host disease [29]. Remarkably, a limited number of studies concerning the polymorphism in MICA gene transmembrane region have been carried out in melanoma disease [30, 31]. However, to date, no studies have been performed on MICA polymorphism in the extracellular domains in melanoma. Thus, the aim of the present study was to determine whether extracellular MICA polymorphism and its previously described linkage disequilibrium with HLA-B locus [22, 32, 33] may influence the susceptibility to cutaneous melanoma in a group of patients from Murcia, a Mediterranean Spanish Region.

## 2. Materials and Methods

### 2.1. Subjects

A total of 233 Caucasian patients with histological diagnosis of cutaneous malignant melanoma were recruited between 1996 and 2013 from the Dermatology Department of University Clinical Hospital Virgen de la Arrixaca in Murcia, Spain. Patient classification was made according to the American Joint Committee on Cancer (AJCC) recommendations for cutaneous melanoma [34]. Melanoma patient age was 53 ± 15 (mean ± SD) years old (112 men and 121 women). Exclusion criteria were history of another malignant, autoimmune, inflammatory, or infectious chronic disease, or immunodeficiency. Additionally, a series of 200 sex- and age-matched Caucasian healthy individuals of the same ethnic origin was also studied. This series consisted of 92 men and 108 women, aged 51 ± 17 (mean ± SD). Demographic data of the enrolled subjects are summarized in Table 1.

All individuals included in the present study were unrelated and randomly selected. In all cases, informed consent was obtained and the protocol of study was approved by the institutional ethical committee, in accordance with the Declaration of Helsinki Principles.

### 2.2. Sample Collection and DNA Preparation

Blood samples were obtained by venipuncture from patients and healthy individuals and collected in anticoagulated vacutainer tubes (Becton Dickinson, Mountain View CA, USA). Genomic DNA was extracted by using the QIAamp DNA Blood Midi Kit (QIAGEN, Hilden, Germany), as recommended by the manufacturer.

### 2.3. MICA and HLA-B Genotyping

The samples were typed for MICA and HLA-B alleles using the polymerase chain reaction-specific oligonucleotide sequence (PCR-SSO) method by microbeads array luminex technology (One Lambda, CA). Reactions were read with the LABScan 100 flow cytometry analyzer and assignment of allelic types was performed using the HLA Fusion 2.0 software (One Lambda, CA). Data of MICA genotyping were also used to classify patients and controls based on the presence of valine (val) or methionine (met) amino acids at codon 129 of the alfa-2 domain.

### 2.4. Statistical Analysis

Demographic data and analysis results were collected in a database (Microsoft Access 11.0; Microsoft corporation, Seattle, WA), and statistical analysis was performed using SPSS 15.0 software (SPSS Inc., Chicago IL, USA). Chi-square test and two-tailed unpaired *t*-test were used to detect differences regarding sex and age, respectively. MICA allele frequencies were estimated by direct counting and represent the percentage of individuals positive for a certain allele. Associations between MICA gene diversity and melanoma disease were established by comparing MICA allele frequencies in groups of patients with each other, and with healthy controls, using chi-square and two-sided Fisher's exact tests. Data were also analyzed by using a multivariate logistic regression model. Odds ratio (OR) and its 95% confidence interval (CI) were calculated to estimate any relative risk. *P* value <0.05 was considered significant. Corrected *P* value (Pc) was obtained by multiplying the *P* value by the number of alleles tested according to Bonferroni's correction [35]. The power of the statistical analysis was calculated by Post-hoc Power calculator as described (http://clincalc.com/stats/power.aspx).

## 3. Results

### 3.1. Clinical and Histological Characteristics of Patients with Cutaneous Malignant Melanoma

As summarized in Table 1, 162 patients presented histological diagnosis of superficial spreading melanoma (SSM), 29 of nodular melanoma (NM), 16 of lentigo maligna melanoma (LMM), 13 of acral lentiginous melanoma (ALM), 11 of melanoma “*in situ*,” 1 of desmoplastic melanoma (DM), and 1 of spitzoid melanoma (SM).

Patients were also classified according to tumor thickness: ≤1.0 mm and >1.0 mm; the presence or absence of ulceration of primary lesion, and the presence or absence of sentinel lymph node (SLN) metastasis at diagnosis. Data about tumor thickness and about ulceration were available for 232 and 217 out of 233 patients, respectively, while information about SLN biopsy was available for 141 out of 233 patients, 119 of them showing benign SLN (Table 1).

### 3.2. MICA and Melanoma Disease

The frequency of MICA alleles in melanoma patients compared with a control group is summarized in Table 2. MICA^*^009 allele frequency was found to be significantly increased in patients compared with controls (*P* = 0.002, OR = 2.14; 95% CI, 1.31–3.49; Post-hoc Power = 92%); this association remained statistically significant after *P*-correction for multiple testing (Pc = 0.03). When the two most frequent histological subtypes of melanoma, such as SSM and NM, were individually analyzed, a significant increase in the frequency of MICA^*^009 allele was observed in both groups of patients compared with controls (25% in SSM and 35% in NM versus 15% in controls; *P* = 0.016 and *P* = 0.015, resp.).

An additional analysis was performed based on the previous linkage disequilibrium reported between MICA^*^009 and HLA-B^*^51 alleles [22, 32, 33]. In a first analysis this study showed an association between MICA^*^009 and HLA-B^*^51 in patients and control group (Table 3). This association was stronger in the patients because 50% of MICA^*^009 positive patients coexpressed HLA-B^*^51 against 41% of MICA^*^009 positive controls. In fact, the frequency of individuals carrying MICA^*^009 and HLA-B^*^51 was significantly higher in patients than in control group (13%* versus* 6%; *P* = 0.015), as shown in Table 3. However, when a multivariate logistic regression analysis was performed, the results showed that neither MICA^*^009 nor the combination MICA^*^009/HLA-B^*^51 was associated with melanoma susceptibility (Table 4).

On the other hand, a decreased frequency of MICA^*^002 was found in melanoma patients compared with controls (*P* = 0.004, Pc = 0.07; OR = 0.50, 95% CI, 0.32–0.80; Post-hoc Power = 79%), as shown in Table 2. Likewise, MICA^*^010 and MICA^*^019 frequencies were also underrepresented in patients compared with controls (*P* = 0.022, Pc = 0.37; OR = 0.27, 95% CI, 0.09–0.86; Post-hoc Power = 58% and *P* = 0.014, Pc = 0.24; OR = 0.10, 95% CI, 0.01–0.83; Post-hoc Power = 74%, resp.), but significances were lost after *P*-correction.

The distribution of MICA allele frequencies according to the dimorphism at position 129, and their genotypes, was also analyzed, but no significant differences were found between patients and controls, as shown in Table 5.

The influence of MICA polymorphism on melanoma was further evaluated according to clinical findings at diagnosis such as tumour thickness, ulceration of primary lesion, and SLN metastasis, but no significant differences were observed comparing groups of patients with each other, nor when each group of patients was compared with controls (data not shown).

## 4. Discussion

In the present work, we have investigated for the first time the influence of MICA polymorphism in the extracellular domains on melanoma disease in a group of patients with cutaneous malignant melanoma from southeastern Spain. A total of 17 MICA alleles were detected (see Table 2). MICA allele frequencies found in our control series are similar to those previously reported in other Caucasian populations [22, 36, 37]. Moreover, a number of positive and negative associations with specific MICA alleles were revealed by comparing melanoma patients with healthy individuals. In a first analysis, we found a positive association of MICA^*^009 allele with cutaneous malignant melanoma. Immunologically related studies such as those investigating Behçet's disease also found a positive association between MICA^*^009 and disease development [26, 36, 37]. However, in these studies a relationship between HLA-B^*^51 and Behçet's disease was also reported, suggesting that the observed association between MICA^*^009 and Behçet's disease arises from a strong linkage disequilibrium with HLA-B^*^51.

Thus, based on the previous linkage disequilibrium reported between MICA^*^009 and HLA-B^*^51 alleles [22, 32, 33], a further analysis was performed in which we found a high association between MICA^*^009 and HLA-B^*^51 in both the control and patient groups. This association was stronger in patients than in the control group. Nonetheless, a multivariate logistic regression model showed that neither MICA^*^009 nor the combination MICA^*^009/HLA-B^*^51 was associated with melanoma susceptibility.

Other findings in this study include the decreased frequency of MICA^*^002 on melanoma disease. The protective effect of MICA^*^002 observed in melanoma disease was also reported in other pathologies like primary sclerosing cholangitis [38]. Nevertheless, studies with larger series are required to find out whether MICA^*^002 allele may result in protection from cutaneous melanoma.

On the other hand, no differences were found in the genotype distribution of affinity variants between melanoma patients and controls, unlike what was observed in other pathologic settings [25, 29, 37, 39], where MICA-129 genotype could be considered as a biomarker of disease susceptibility and/or protection.

In accordance with our data, other case-control studies were not able to identify an association between MICA gene polymorphism and the occurrence of melanoma skin cancer, although in this case the polymorphism was studied at transmembrane level [30, 31]. In addition, multiple genome-wide association studies (GWAS) in melanoma patients have identified some genetic variants associated with an increased risk of melanoma development [40–42]. However, these variants are located in several regions (16q24, 11q14-q21, 9p21, 1q21.3, and 20q11.22), other than the region where the MICA gene is placed (6p21.3). All these studies, as well as ours, have failed to establish an association between MICA gene polymorphism and an increased risk of developing cutaneous malignant melanoma.

In conclusion, our data demonstrate a lack of association between MICA polymorphism in the extracellular domains and cutaneous malignant melanoma, at least in our population.

## Figures and Tables

**Table 1:** Demographic data and histological and clinical characteristics of melanoma patients at diagnosis.

Demographic data	Controls *N* = 200	Melanoma patients *N* = 233
Age (years)^a^	51 ± 17	53 ± 15
Gender		
Male	92	112
Female	108	121

Histological and clinical characteristics		*N* (%)

Histological subtypes		
SSM		162 (69)
NM		29 (12)
LMM		16 (7)
ALM		13 (6)
Melanoma “*in situ*”		11 (5)
DM		1 (0.5)
SM		1 (0.5)
Clinical characteristics		
Tumor thickness		
≤1 mm		128 (55)
>1 mm		104 (45)
Ulceration at primary lesion		
Yes		44 (20)
No		173 (80)
SLN metastasis		
Yes		22 (16)
No		119 (84)

SSM: superficial spreading melanoma; NM: nodular melanoma; LMM: lentigo maligna melanoma; ALM: acral lentiginous melanoma; DM: desmoplastic melanoma; SM: spitzoid melanoma; SLN: sentinel lymph node. ^a^Mean ± SD.

**Table 2:** Allelic frequency of MICA gene in melanoma patients and controls.

Allele	Controls *N* = 200 *n* (*%*)	Melanoma patients *N* = 233 *n* (*%*)	*P*/Pc value
MICA^*^001	24 (*12*)	38 (*16*)	
MICA^*^002	57 (*28*)	39 (*17*)	0.004/0.07
MICA^*^004	52 (*26*)	77 (*33*)	
MICA^*^005	1 (*0.5*)	0 (*0*)	
MICA^*^006	1 (*0.5*)	0 (*0*)	
MICA^*^007	12 (*6*)	14 (*6*)	
MICA^*^008	105 (*52*)	117 (*50*)	
MICA^*^009	29 (*14*)	62 (*27*)	0.002/0.03
MICA^*^010	12 (*6*)	4 (*2*)	0.022/0.37
MICA^*^011	17 (*8*)	23 (*10*)	
MICA^*^012	5 (*2*)	7 (*3*)	
MICA^*^015	7 (*3*)	2 (*1*)	
MICA^*^016	14 (*7*)	26 (*11*)	
MICA^*^017	6 (*3*)	11 (*5*)	
MICA^*^018	11 (*5*)	17 (*7*)	
MICA^*^019	8 (*4*)	1 (*0.4*)	0.014/0.24
MICA^*^027	2 (*1*)	0 (*0*)	

MICA, major histocompatibility complex class I chain-related gene A. *P* value was determined by two-sided Fisher's exact test. *Note.* Valine 129 positive alleles: MICA^*^004, ^*^005, ^*^006, ^*^008, ^*^009, ^*^010, ^*^016, ^*^019, ^*^027; methionine 129 positive alleles: MICA^*^001, ^*^002, ^*^007, ^*^011, ^*^012, ^*^015, ^*^017, ^*^018.

**Table 3:** Association between MICA^*^009 and HLA-B^*^51 in melanoma patients and controls.

MICA^*^009	HLA-B^*^51	Controls *N* = 200 *n* (*%*)	Melanoma patients *N* = 233 *n* (*%*)	*P *value
+	+	12 (*6*)	31 (*13*)	0.015
+	−	17 (*8.5*)	31 (*13*)	
−	+	5 (*2.5*)	6 (*3*)	
−	−	166 (*83*)	165 (*71*)	
*P* value		<0.0001	<0.0001	

Association between MICA^*^009 and HLA-B^*^51 in melanoma patients and controls. MICA: major histocompatibility complex class I chain-related gene A; HLA: human leukocyte antigen. *P* value was determined by two-sided Fisher's exact test.

**Table 4:** Multivariate logistic regression analysis for factors which could influence melanoma development.

	OR	95% CI	*P *value
Age	1.01	0.99–1.02	0.29
Sex	0.94	0.64–1.38	0.75
MICA^*^009	1.76	0.93–3.31	0.082
MICA^*^009/HLA-B^*^51	1.42	0.58–3.47	0.44

Multivariate logistic regression analysis of the effect of MICA^*^009 and MICA^*^009/HLA-B^*^51 combination on melanoma susceptibility.

MICA: major histocompatibility complex class I chain-related gene A; HLA: human leukocyte antigen; OR: odds ratio; CI: confidence interval.

*Note*. Comparisons were made between the total of patients and the control group as the reference group.

**Table 5:** MICA-129 allele and genotype frequencies in melanoma patients and controls.

MICA-129 polymorphism	Controls *N* = 200 *n* (%)	Melanoma patients *N* = 233 *n* (%)	*P* value
Alleles			
Val^129^	177 (*88*)	209 (*90*)	0.76
Met^129^	122 (*61*)	131 (56)	0.33
Genotypes			
Val/Val^129^	78 (*39*)	102 (*44*)	0.33
Met/Val^129^	99 (*49*)	107 (*46*)	0.50
Met/Met^129^	23 (*12*)	24 (*10*)	0.76

MICA: major Histocompatibility complex class I chain-related gene A. *P* value for disease susceptibility was determined by two-sided Fisher's exact test.

*Note*. Valine 129 (Val^129^) positive alleles: MICA^*^004, ^*^005, ^*^006, ^*^008, ^*^009, ^*^010, ^*^016, ^*^019, ^*^027; methionine 129 (Met^129^) positive alleles: MICA^*^001, ^*^002, ^*^007, ^*^011, ^*^012, ^*^015, ^*^017, ^*^018.

## Abbreviations

MHC: Major histocompatibility complex
MIC: Major histocompatibility complex class I chain-related gene
MICA: Major histocompatibility complex class I chain-related gene A
HLA: Human leukocyte antigen
PCR: Polymerase chain reaction.
